# Seasonality of climatic drivers of flood variability in the conterminous United States

**DOI:** 10.1038/s41598-019-51722-8

**Published:** 2019-10-25

**Authors:** Jesse E. Dickinson, Tessa M. Harden, Gregory J. McCabe

**Affiliations:** 1 U.S. Geological Survey, Tucson, Arizona USA; 2 U.S. Geological Survey, Portland, Oregon USA; 3 U.S. Geological Survey, Denver, Colorado USA

**Keywords:** Hydrology, Hydrology

## Abstract

Flood variability due to changes in climate is a major economic and social concern. Climate drivers can affect the amount and distribution of flood-generating precipitation through seasonal shifts in storm tracks. An understanding of how the drivers may change in the future is critical for identifying the regions where the magnitude of floods may change. Here we show the regions in the conterminous U.S. where seasonal changes in global-scale climate oscillations have driven a large part of the variability of flood magnitude. The regions are cohesive across multiple watershed boundaries suggesting that variability in floods is driven by regional climate influences. Correlations with climate indices indicate that floods in the western and southern U.S. are most affected by global-scale climate. The regions provide a useful approach for characterizing flood variability and for attributing climatic drivers on flood variability and magnitude.

## Introduction

Flood variability in a changing climate is a major economic and social concern. To complement our understanding of future flooding, many studies have focused on trends, frequency, and the nonstationarity of floods^1–7^ because of climate and land surface changes^5,8–14^. Such changes can have compounding effects on floods that vary regionally and through time, which makes it difficult to detect and quantify the importance of multiple drivers on floods^15^. An understanding of these drivers is critical because of possible shifts of the hydrologic cycle in a future climate^16–19^.

**Figure 1:**
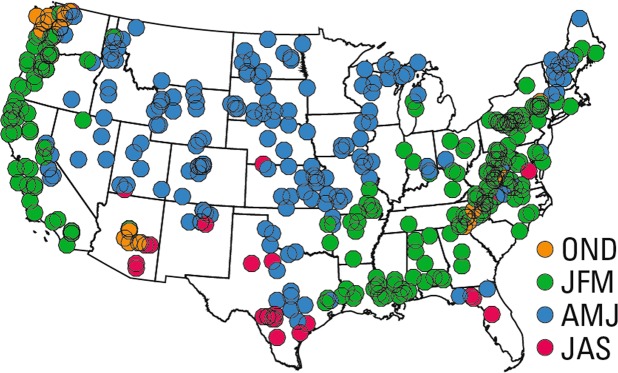
Mean season of the annual peak flow from 1966-2015 at 415 streamflow gauges in the USGS HCDN-2009 network. The seasons are defined as October-December (OND), January-March (JFM), April-June (AMJ), and July-September (JAS).

Here we show the regions in the conterminous U.S. where seasonal changes in global-scale climate drive part of the variability of flood magnitude^13,20,21^. Flood variability is often related to regional shifts in seasonal atmospheric pathways of moisture delivery^20,22^. The seasonality of floods (as the mean day of flood peaks) in the U.S. has been found, with few exceptions, to have little signal of temporal change^23^, and generally happens between January and June over much of the conterminous U.S. (Fig. 1). In a regional sense, seasonal mean streamflow variability generally groups into hydroclimatic regions more so than with watersheds^24^. Within such regions, mean annual streamflow is significantly correlated with at least one well-known climate index^24^ (for example, the El Nino Southern Oscillation (ENSO)^25^, the Pacific Decadal Oscillation (PDO)^26^, the Pacific North American Index^27^ (PNA), the Atlantic Multidecadal Oscillation^28^ (AMO), the North Atlantic Oscillation^29^ (NAO), and the Arctic Oscillation (AO)^30^). For these reasons, we used a regional approach to investigate whether seasonal variations in global circulation are key factors for regional and temporal variability of floods in the conterminous U.S.

## Regional Flood Variability

We used a cluster analysis of 415 streamflow gauge records in the U.S. Geological Survey Hydro-Climatic Data Network 2009 network^31^ (HCDN-2009) (which represents minimally altered watersheds) to identify regional patterns in the magnitude of annual and seasonal flood variability (see methods for more details). We examined a 50-year record (1966–2015) of annual peak (maximum instantaneous discharge during a water year between October 1 and September 30) and seasonal maximum flows to represent floods. The seasons are defined as October through December (OND), January through March (JFM), April through June (AMJ), and July through September (JAS).

**Figure 2:**
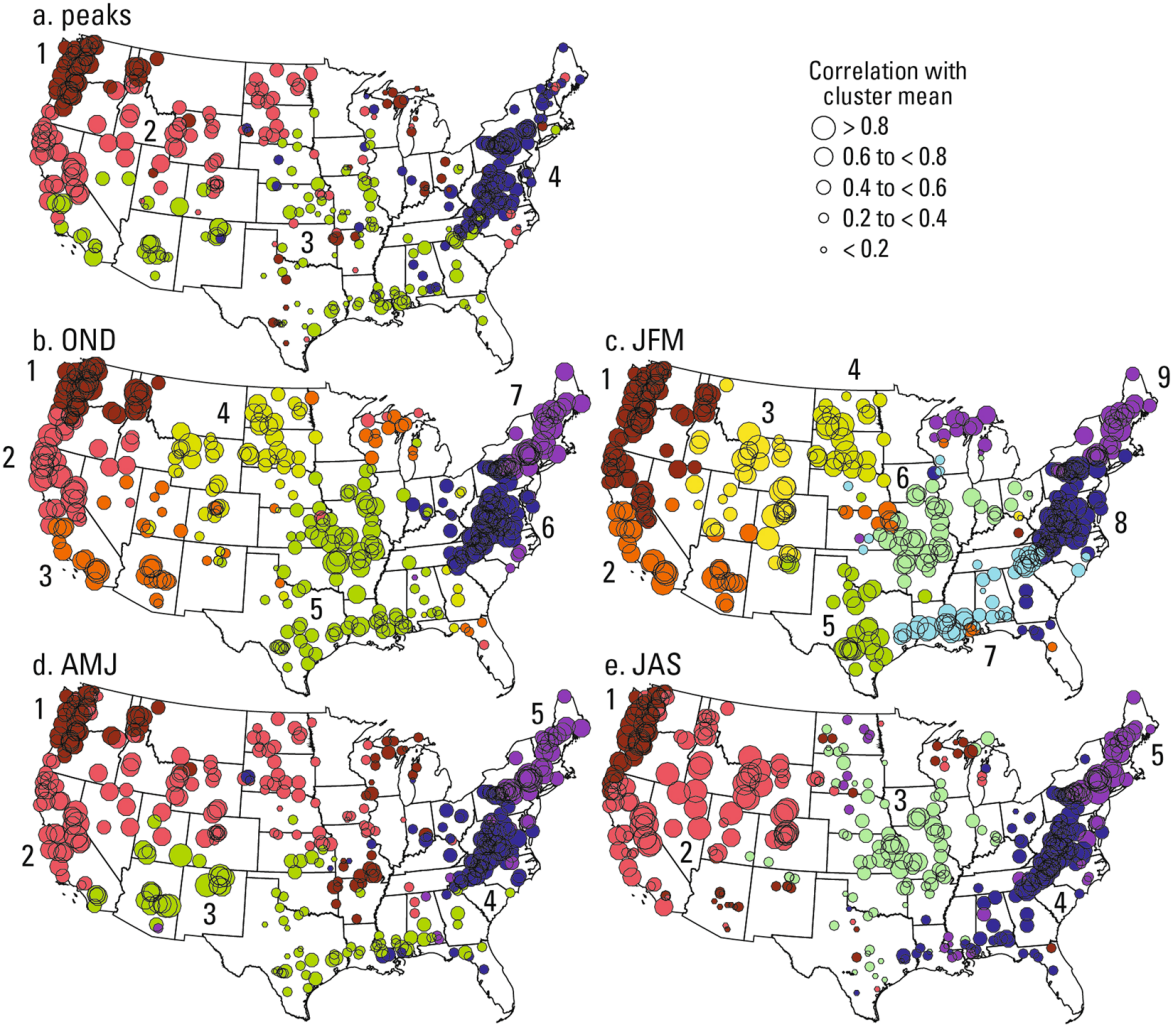
Maps showing time series clusters of (**a**) annual peak flows and maximum daily flow for the four seasons: (**b**) October through December (OND), (**c**) January through March (JFM), (**d**) April through June (AMJ), and (**e**) July through September (JAS) from 1966 to 2015.

The gauges clustered into spatially coherent geographic regions based solely on the flow data (Fig. 2). The gauges are clustered into four regions based on the annual peaks, seven regions based on OND maximum flows, nine regions for the JFM maximum flows, and five regions each for AMJ and JAS maximum flows. The regions are arbitrarily numbered from the northwestern to northeastern U.S. The size of the circles in each cluster in Fig. 2 indicate the level of correlation between the peak and the seasonal-maximum flow at the gauge and cluster-mean flow (mean of peak and seasonal-maximum flow at all gauges in the cluster). That is, larger circles represent gauges with flow variability that strongly resembles the variability at other gauges in the cluster, whereas gauges with smaller circles have less similarity of flow variability to other gauges within its cluster. Table 1 provides statistical summaries for the clusters and the *p* value for the Mann-Kendall non-parametric trend test for a monotonic trend in the cluster-mean flow for each cluster.

**Table 1:** The mean, maximum (max), minimum (min) correlation coefficient *r* between the mean time series for the peaks and seasonal-maximum flow clusters and the same flows at gauges within the clusters, the count of gauges in each cluster, and the *p* value for the Mann-Kendall non-parametric trend test for a monotonic trend in the mean-cluster time series (*p* < 0.05 in bold).

	cluster									
	statistic	1	2	3	4	5	6	7	8	9
peaks	mean *r*	0.46	0.51	0.36	0.44	—	—	—	—	—
	max *r*	0.86	0.87	0.75	0.82	—	—	—	—	—
	min *r*	0.09	0.09	0.05	0.16	—	—	—	—	—
	count	73	99	125	114	—	—	—	—	—
	MK *p*	0.78	0.39	0.12	0.81	—	—	—	—	—
OND	mean *r*	0.74	0.70	0.53	0.50	0.51	0.64	0.70		
	max *r*	0.90	0.93	0.83	0.82	0.82	0.87	0.86	—	—
	min *r*	0.57	0.31	0.21	0.15	0.14	0.34	0.15	—	—
	count	40	44	52	65	94	84	36	—	—
	MK *p*	0.20	0.50	**0.03**	0.11	0.19	1.00	0.10	—	—
JFM	mean *r*	0.66	0.69	0.64	0.66	0.67	0.61	0.56	0.62	0.63
	max *r*	0.88	0.92	0.91	0.90	0.91	0.85	0.81	0.88	0.89
	min *r*	0.29	0.30	0.22	0.32	0.35	0.01	0.19	0.19	0.11
	count	72	41	43	28	26	40	47	78	40
	MK *p*	0.55	0.42	0.17	0.79	0.89	0.87	0.53	0.49	0.96
AMJ	mean *r*	0.50	0.52	0.46	0.49	0.61	—	—	—	—
	max *r*	0.80	0.89	0.86	0.81	0.88	—	—	—	—
	min *r*	0.03	0.15	0.11	0.02	0.24	—	—	—	—
	count	72	121	89	88	45	—	—	—	—
	MK *p*	0.78	0.87	**0.03**	0.59	1.00	—	—	—	—
JAS	mean *r*	0.51	0.63	0.41	0.51	0.54	—	—	—	—
	max *r*	0.91	0.92	0.85	0.87	0.88	—	—	—	—
	min *r*	0.11	0.05	0.04	0.07	0.09	—	—	—	—
	count	67	94	85	108	56	—	—	—	—
	MK *p*	**0.01**	0.08	0.84	0.69	**0.02**	—	—	—	—

The ‘—’ indicates no correlation test.

We found that the spatial patterns of the clusters correspond more to hydroclimatic regions related to atmospheric moisture delivery rather than regional drainage-basin boundaries, which is similar to previous findings for mean annual streamflow^24,32^. A distinct pattern in the northwestern U.S. (cluster 1) for annual peaks and all seasonal maximum streamflow corresponds to a relatively consistent pathway of moisture delivery from the North Pacific Ocean^33–35^. That is, variations in moisture delivery may drive part of the variability of floods in this region. Cluster patterns for the central eastern coast of the U.S. and Appalachian Mountain regions are consistent with findings that this region receives moisture mainly from the Atlantic Ocean and Gulf region^33^.

The spatial patterns and number of clusters for both annual and seasonal maximum streamflow vary for the southwest, southeast, and central U.S., which suggests that seasonal shifts in atmospheric moisture delivery^35^ are important for peaks and seasonal maximum flows in those regions than other regions indicated by the clusters. The seasonal variability of cluster patterns in the central U.S. are likely related to seasonal shifts in the pathways of flood-generating moisture from the Pacific and Atlantic Oceans and Gulf of Mexico^20^. For JFM a distinct cluster includes much of the southeastern U.S. and may be related to the variability of moisture from the Gulf of Mexico and Atlantic Ocean that is limited to the Gulf Coast region in winter. For AMJ the distinct southeast cluster found for the JFM data shifts to the southwest, and the AMJ maximum flows resemble the annual peak flows across the U.S. For JAS, the spatial pattern of clusters shifts toward large regions for the central and southeast U.S. that may be related to the variability of widespread moisture from the Gulf of Mexico and Atlantic Ocean that intrudes into the southern U.S.^20^. The spatial patterns of clusters in the southwestern U.S. likely vary because of seasonal differences in moisture delivery (frontal systems, monsoon-driven, and tropical storms) from the Pacific. The seasonal differences in the spatial patterns of the clusters demonstrate that seasonal variability of moisture sources produces different regions of peak flow variability

## Flood Variability through Time

**Figure 3:**
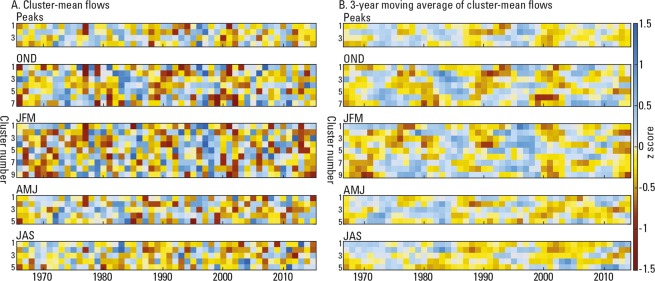
(**A**) Cluster-mean flows from 1966–2015 and (**B**) their three-year moving averages of annual peaks and seasonal-maximum flows from 1967 to 2014. The time series are grouped by peaks and season and arranged horizontally in each group by cluster number. The year is incremented vertically along the y axis. Years with larger flows are shown in blue and years of lower flows are shown in yellow and brown.

We found that the magnitude of peaks and seasonal maximum flows were highly variable year-to-year from 1966 to 2015 (Fig. 3) but the variability had some temporal persistence. The annual time series of the cluster-mean flows appear nearly random, so we used a three-year moving average to retain interannual oscillations. For the annual peaks, the smoothed time series indicate periods of lower-than-average peaks (yellow and red) in almost all clusters before 1970, in the mid-to-late 1980’s, and generally between 2000 to 2015, especially during several years around 2000. Periods of higher annual peaks (blue shades) occurred nationwide during the early to mid-1970’s, early to mid-1980’s and the mid-to-late 1990’s.

Many of the temporal patterns in the seasonal maximum flow metrics resemble the variations in annual peak flows. With few exceptions, below-average maximum flows prior to 1970 (1966–1969) occurred nationwide in OND and JFM—in AMJ and JAS below-average flows were only in clusters 4 and 5 (southeast and eastern U.S.). Above-average maximum flows were highest during OND and AMJ in the early-to-mid 1980’s. Other above-average maximum flows occurred in JAS during that same period in clusters 1 and 2 (western U.S.) while the Midwest and eastern U.S. experienced below-average maximum flows. Above-average flows in the mid-to late 1990’s mostly occurred during OND and JFM. Since 2000, the tendency of below-average maximum flows is more prevalent during AMJ and JAS until about 2012 when OND and especially JFM also experienced below-average maximum flows. The below-average flows in the early 2000s generally occurred in all seasons.

We used trend tests and frequency analyses to examine temporal persistence in the cluster-mean flows (Table 1). A Mann-Kendall^36^ trend test on the unsmoothed cluster-means found significant trends at *p* < 0.05 only for region 3 (southwestern U.S.) in the OND and AMJ seasons, and in region 1 for the JAS season (northwestern U.S.). A lack of significant trends in large flows is consistent with previous findings^1,7,37–39^. To identify periodic variability, we used a Discrete Fourier Transform (DFT) with Hann windowing^40^ and Morlet wavelet analysis^41^ of the cluster-mean flows (Supplementary Figs 2–6) and their respective 3-year moving averages (Supplementary Figs 7–11). Using the DFT, none of the raw (unsmoothed) cluster-mean flows had periodicities (*p* < 0.1 against a red noise null hypothesis). The 3-year moving averages showed several significant periodicities using DFT (not shown in Figures): 6–7 years (*p* < 0.05) in cluster 4 (eastern U.S.) annual peak flows; 9–10 years (*p* < 0.1) and 6 years (*p* < 0.05) in clusters 2 (west coast) and 3 (southwestern U.S) OND maximum flows; 6–7 years (*p* < 0.05) in clusters 4 (northern plains) and 8 (eastern U.S.) JFM maximum flows; 5 years (*p* < 0.1) in cluster 3 (southern U.S.) AMJ maximum flows; and 7–8 years (*p* < 0.1) in cluster 1 (northwestern U.S.) JAS maximum flows. The wavelet analysis of the annual data indicated little signal of periodicity (Supplementary Figs 2–6). In the 3-year moving averages (Supplementary Figs 7–11) we identified some signals of variability of around 4–7 years and a decadal band of 10–16 years. The higher frequency (4–7 years) variations were mostly intermittent in explanatory power between successive years from 1967–2014 for all clusters and generally matches ENSO variability^42,43^. The decadal band (10–15 years) was more common in clusters in the western U.S. This band resembles variations in western U.S. streamflow that were coherent with ENSO variability^44^.

## Climatic Drivers of Annual and Seasonal Floods

**Figure 4:**
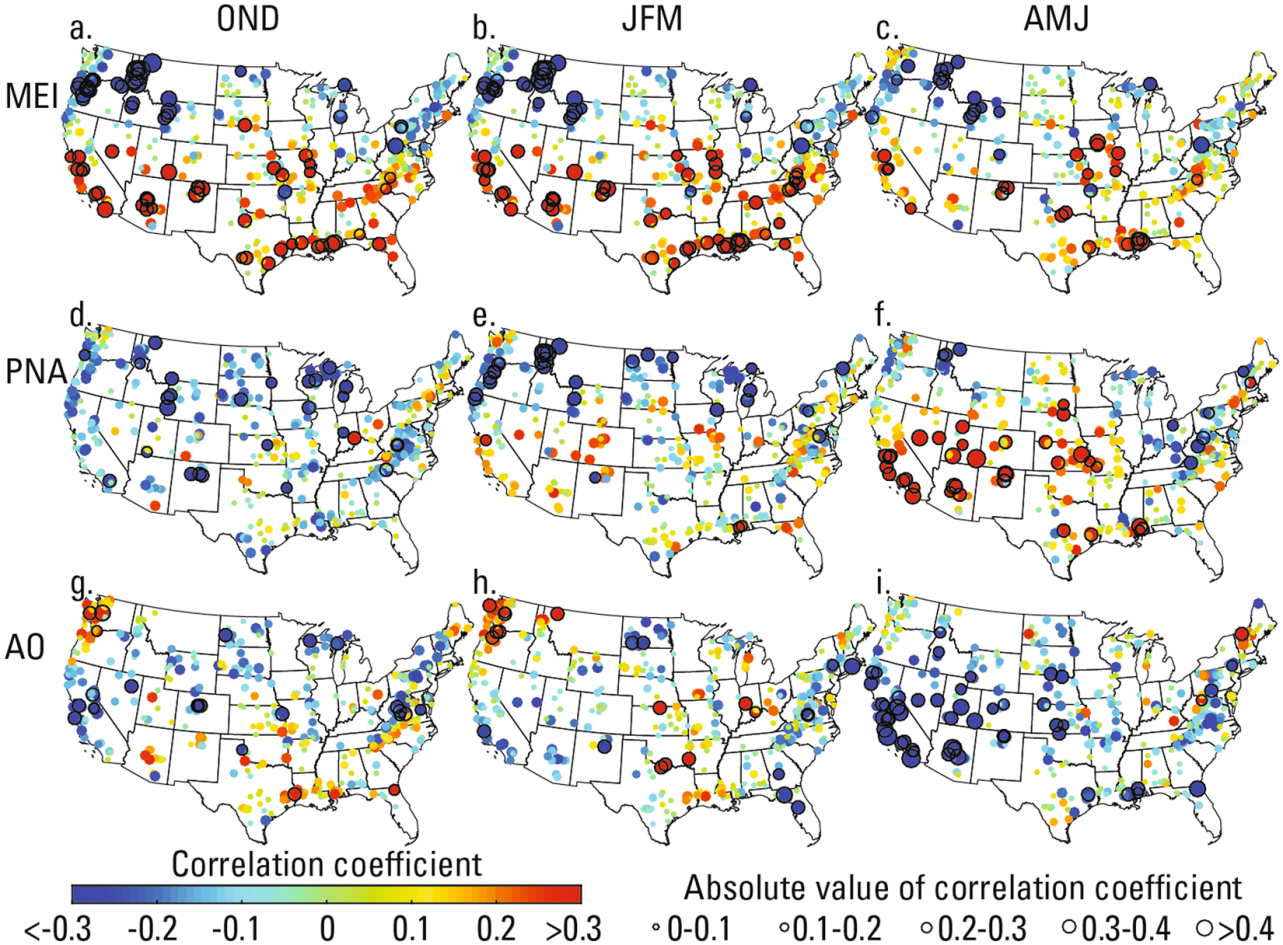
Correlation of peaks at gauges with mean climate indices for months OND, JFM, AMJ. With MEI in (**a–c**), PNA in (**d–f**), and AO in (**g–i**). The color and size of the marker indicates the value of the correlation coefficient. Markers with dark outlines indicate significance at *p* < 0.05.

The regional clusters have some correspondence with the spatial relations of precipitation with climate indices, particularly in the western U.S.^27,45,46^. So, we examined the relations between climate indices and the cluster-mean flows using Pearson correlation. Because the individual gauge records are averaged for each cluster, we further examined the relations of the seasonal means of the climate indices with annual peaks (Fig. 4) and with seasonal maximum streamflow (Supplementary Fig. S1) at each gauge. We also correlated the cluster-mean flows with mean water-year sea surface temperature (SST) (Supplementary Figs S12–S16). The climate indices were the MEI^47^, PDO^26^, PNA^27^, AMO^28^, NAO^29^, and AO^30^. Table 2 shows significant correlations (*p* < 0.05) between annual peaks and seasonal means of the climate indices in bold and in outlined cells and greater significance (*p* < 0.01) as bold text in outlined cells. The clusters with field significance (*p* < 0.05) are indicated by a percentage in bold (see methods for details). Figure 4 shows correlations of the seasonal climate indices and peak flows at individual gauges that were selected for having many significant correlations over coherent regions.

**Table 2:** Correlations between the cluster-mean time series for peaks and seasonal-mean climate indices, and the percentage of gauges in each cluster with significant correlation with the indices (percentage is bold if passed the field significance test). Correlations of the mean time series that are significant at the 95% confidence level are in bold and outlined cells.

	cluster				
	climate index	1	2	3	4
OND	MEI	−**0.37 29%**	−0.11 13%	**0.57 34%**	−0.07 1%
	PDO	−**0.34 22%**	−0.14 6%	0.23 6%	0.15 3%
	PNA	−0.23 8%	−0.23 7%	−0.20 6%	−0.13 6%
	AMO	0.21 14%	0.03 4%	−**0.32** 10%	−0.02 12%
	NAO	−0.13 4%	−**0.34 19%**	0.17 8%	−0.18 10%
	AO	0.14 4%	−**0.28** 10%	0.12 2%	−0.17 8%
JFM	MEI	−**0.31 25%**	−0.11 11%	**0.61 36%**	0.00 3%
	PDO	−0.24 14%	0.00 5%	0.17 4%	0.09 4%
	PNA	−**0.29 21%**	−0.14 10%	0.13 3%	−0.06 3%
	AMO	0.02 5%	0.09 8%	−0.12 6%	−0.09 10%
	NAO	0.25 12%	−0.10 4%	−0.10 6%	−0.12 4%
	AO	0.27 11%	−0.11 3%	−0.12 6%	−0.11 4%
AMJ	MEI	−0.17 15%	−0.11 9%	**0.39 21%**	−0.01 3%
	PDO	−0.10 3%	0.08 5%	0.24 5%	0.11 4%
	PNA	−0.18 5%	0.11 4%	**0.42 26%**	−0.11 5%
	AMO	−0.19 15%	0.01 7%	0.00 5%	−0.12 4%
	NAO	−0.05 5%	−0.19 9%	−0.20 4%	0.00 4%
	AO	−0.02 0%	−0.28 **17%**	−**0.44 24%**	−0.10 4%
JAS	MEI	0.12 3%	−0.01 3%	0.02 4%	−0.01 0%
	PDO	0.12 4%	0.03 0%	0.27 **12%**	−0.04 5%
	PNA	0.09 1%	−0.01 0%	0.15 4%	−0.08 3%
	AMO	−0.13 12%	−0.09 2%	−0.12 4%	−0.08 4%
	NAO	0.14 4%	0.13 1%	0.12 2%	0.16 7%
	AO	−0.14 1%	0.00 3%	−0.01 5%	−0.08 2%

Correlations that are significant at the 99% confidence level are shown in bold outlined cells.

Peak flows in the northwestern U.S. (cluster 1) and southern and central U.S. (cluster 3) were significantly correlated with the most climate indices we examined (Table 1). This includes negative correlations of the northwest with the MEI during OND and JFM months, with the PDO in OND, and the PNA pattern in JFM. The southern and central U.S. (cluster 3) had greater significance (positive) with MEI in OND (*p* < 2E-5), JFM (*p* < 3E-6), and AMJ (*p* < 6E-3), and with PNA in AMJ (*p* < 3E-3). These patterns are consistent with the dipole pattern of correlations of precipitation^45,48^ and streamflow with ENSO^49^, PDO, and PNA^27^, in which precipitation is less than normal in the northwestern U.S. and greater than normal in the southwest and southern U.S. during positive phases of MEI, PDO, and PNA. These correlations with peaks and MEI, PDO, and PNA are stronger (more negative in the northwest, and more positive in the central and southern U.S.) than those identified for mean annual streamflow^24^. The southern and central U.S. also had greater significance (negative) with AO in AMJ (*p* < 1E-3), which may be related to the relations between the Northern Annular Mode (indicated by NAO and AO) and storm tracks across the western U.S. and northern central plains^50^. Cluster 2 (western U.S. and central plains) also had significant correlation (negative) with AO in AMJ for the same reasons and in OND months possibly because of changes in the strength of the westerlies^51^. The southern and central U.S. (cluster 3) had significant negative correlations with the AMO in OND, which may be related to negative correlations of AMO and precipitation in similar areas^52^. Somewhat surprising, cluster 4, which includes most of the northeastern U.S., was not significantly correlated to any of the climate indices we investigated.

Like the peak flows, the seasonal cluster-mean maximum flows in the western and southern U.S. were significantly correlated with the most climate indices (Supplementary Table S1). Supplementary Fig. S1 shows correlations between all seasonal climate indices investigated here and the seasonal maximum flows at individual gauges. The cluster-mean correlations using the seasonal maximum flows were sometimes greater than those for the peak flows, likely because the smaller sizes of the seasonal clusters could better represent local teleconnections. The correlations with seasonal indices may also represent teleconnections during specific seasons. Correlations of peaks with the seasonal mean of climate indices may not be as clear because the peaks can occur anytime in a water year, whereas the climate index was determined for a fixed season. Additional correlations revealed through the seasonal analysis (rather than with the peaks) include the eastern U.S. (cluster 6) with PNA in OND (negative, *p* < 6E-3), the south-central U.S. (cluster 5) with PNA (positive) and western U.S. (cluster 3) with NAO in JFM (positive). In JAS, significant correlations include the northeastern U.S. with PDO (negative), the central U.S. with AO (negative, *p* < 5E-3) and the southeastern U.S. with NAO (negative, *p* < 2E-3) and AO.

## Implications for flood attribution

Here we identified regions in the conterminous U.S. where variations in flood magnitude can be largely attributed to seasonal global-scale climate drivers. These drivers can affect the amount and distribution of flood-generating precipitation through shifts in storm tracks. An understanding of how the drivers may change in a future climate^53–55^ may be critical for identifying the regions where the magnitude of floods may change. For example, an enhanced hydrologic cycle may increase the importance of moisture deliveries to North American that are related to ENSO^56^. Peak flows in clusters 1 and 3 (Fig. 2, northwest and southern U.S.) and seasonal maximum flows at many gauges in the northwest, southern, and eastern regions of the U.S. were significantly correlated with MEI. Intensification of moisture related to ENSO may result in changes in flood variability in those regions. It is possible that shifts in climatic variability may result in a different distribution, timing, and amount of precipitation, but these shifts themselves and their effects on the hydrologic cycle are uncertain^53,57^. Using the period of record from 1966–2015, seasonal climate drivers (as indicated by MEI, PNA, and AO) explain much of the flood variability in the western and southern U.S. Changes in the peak flows and the seasons with the largest flows may be more important for flood studies than those seasons having a lower maximum flow (for example, lower seasonal maximum flows in summer in the northeastern U.S.). Here, we examined standardized flows, which do not indicate flood magnitudes. This analysis provides climate attributions for these regions which may help to disentangle the compounding effects of climate variability on flood attribution studies.

## Methods

### Streamflow data

Streamflow data were obtained from the U.S. Geological Survey (USGS) National Water Inventory System (NWIS, waterdata.usgs.gov) database for a 50-year period for water years (October through September) 1966 to 2015. We selected sites from the HCDN-2009, which is a subset of USGS streamflow gauges that have minimal anthropogenic interference (e.g. minimal effects of dams, diversions, water withdrawals etc.). We selected a subset of 415 gauges from the HCDN-2009 using criteria for completeness of the peak flow record at each stream gauge^7^ (Fig. 1). We also determined a maximum seasonal streamflow for the same 415 gauges for the months October through December (OND), January through March (JFM), April through June (AMJ), and July through September (JAS). For this study, the maximum seasonal streamflow is the largest daily streamflow during the season. The peak flow at a gauge can occur any season, and the mean season of the peak can shift through time^23^. We used circular statistics^58^ to identify the mean day and season of the peak. The mean season of the peak flow is OND in portions of the northwestern and southwestern U.S. as well as part of the Appalachian region, JFM in most of California and the eastern U.S., and AMJ in much of the western interior U.S. The peak occurs in JAS at a few of the analyzed gauges in the southern U.S., mainly parts of Florida and Texas.

### Climate data

Many studies have identified relations between precipitation (and streamflow) and global-scale climatic drivers^45,48,59,60^. To evaluate relations between the peaks, seasonal maximum streamflow and climate, we obtained monthly sea surface temperature (SST) data, monthly atmospheric pressure data for the 500 hecto-Pascal pressure surface, and climate indices for the water years 1966 to 2015. We computed the mean of the climate indices to represent seasonal values for the periods of NDJF (November through February) and for OND, JFM, AMJ, and JAS.

We obtained time series for the Multivariate El Nino Southern Oscillation (ENSO) Index^47^ (MEI), the Pacific Decadal Oscillation^26^ (PDO) index, the Pacific North American (PNA) Index, the Atlantic Multidecadal Oscillation (AMO), the North Atlantic Oscillation (NAO), and the Arctic Oscillation (AO). The MEI index was obtained from the National Oceanic and Atmospheric Administration (NOAA) Earth Systems Research Laboratory (ESRL) Physical Science Division (PSD) (https://www.esrl.noaa.gov/psd/, accessed April 19, 2017). The MEI is measure of the intensity of coupled ocean and atmosphere ENSO processes over the tropical Pacific^47^. The PDO was downloaded from the University of Washington Joint Institute for the Study of the Atmosphere and Ocean (http://research.jisao.washington.edu/pdo, accessed 4-28-2017). The PDO is a multi-decadal ENSO-like pattern of SST variability in the North Pacific defined as the first principal component of monthly SST anomalies over the North Pacific Ocean^43^. The PNA index was downloaded from the NOAA Climate Prediction Center (CPC) (http://www.cpc.ncep.noaa.gov/data/teledoc/telecontents.shtml, accessed 9-20-2018). The PNA^27^ is a measure of atmospheric pressure anomalies over the Pacific Ocean and North America which affect the movement and strength of the East Asian jet stream and is strongly influenced by ENSO oscillations. The AMO was obtained from the NOAA ESRL PSD (https://www.esrl.noaa.gov/psd/data/timeseries/AMO/, accessed 4-19-2017). The AMO^28^ is a measure of SST variability in the North Atlantic Ocean and is related to air temperature and precipitation anomalies in North America. The station-based NAO index was downloaded from the National Center for Atmospheric Research Climate Analysis Section (https://climatedataguide.ucar.edu/climate-data/hurrell-north-atlantic-oscillation-nao-index-station-based, accessed December 11, 2017). The NAO^29^ is a measure of the differences of sea-level pressure between the Subtropical (Azores) High and the Subpolar Low^29^. The AO index^30^ was downloaded from the NOAA CPC (https://www.cpc.ncep.noaa.gov/products/precip/CWlink/daily_ao_index/ao.shtml, accessed 11-9-18). The AO is an index of the variation in the strength of the polar vortex that is casually related to weather patterns in North America.

Monthly sea surface temperature (SST) data were obtained from the Kaplan Extended SST V2 dataset, which contains SSTs gridded at 5° resolution from 87.5°S to 87.5°N and from 2.5°E to 357.5°E (Kaplan *et al*., 1998, www.esrl.noaa.gov/psd/data/gridded/data.kaplan_sst.html, accessed March 17, 2018). Monthly geopotential height (GPH) data at 500 hector-Pascals (h500) gridded at 2.5° resolution from 90°S to 90°N and from 0° to 357.5°E were downloaded from the NOAA ESRL PDS NCEP/NCAR Reanalysis (https://www.esrl.noaa.gov/psd/data/gridded/data.ncep.reanalysis.derived.html, accessed November 9, 2018). We computed water-year averages (October to September) of SST and GPH from 1966 to 2015 at each grid location in the datasets.

### Standardization

The streamflow data were standardized prior to analysis by the procedure for the Standardized Precipitation Index^61,62^. SPI was developed as a means for identifying periods of dryness or wetness in precipitation as a standard normal variable and has become a common approach for quantifying the intensity of drought^63^. SPI is useful for precipitation because it accounts for time series that follow a skewed distribution, and for different amounts of skew between regions with different climates. These properties make SPI a useful approach for quantifying periods of high and low streamflow because annual and seasonal peak flow records are often skewed (usually positively with the tail toward larger flows) and are generated by diverse processes. The SPI procedure provides a means to make comparisons of flood variability across different regions and climates. Standardized streamflows computed using the SPI procedure have a mean of 0 and a variance of 1, which are the same properties obtained from a *z*-score transformation.

### Regionalizing streamflow

Clustering based on a correlation approach^24^ was used to group the gauges into regions with common temporal variability. The clusters were created using a hierarchical cluster analysis of the time series of annual peak and seasonal maximum streamflow for water years 1966 through 2015. The clustering procedure involved a correlation-based approach to identify groups of sites with similar temporal variability of annual peak and seasonal maximum flows (thus five separate clustering procedures were performed: annual peak flows, OND maximum flows, JFM maximum flows, AMJ maximum flows, and JAS maximum flows). The correlation-based clustering process involved the following steps. First, the standardized time series of flows for each site were correlated (using Pearson correlation) with time series of standardized flows for all other sites. The site that was correlated with the most other sites above an arbitrary specified threshold (*r* > 0.5) was removed from the original set of sites along with all the sites that are correlated with the selected site above the specified threshold; these sites initiated the first cluster. To find the second cluster, using the remaining sites, the site that was correlated with the most other sites above the same threshold (*r* > 0.5) was removed along with all the sites that were correlated with the selected sites above the threshold. The process is repeated for the remaining sites to obtain the third and subsequent clusters until there are no remaining sites that have correlations of standardized flows with the other sites above the specified threshold. Sites that were not correlated with another site above the threshold were not assigned to a cluster. A second screening was done to ensure that all sites were assigned to the most representative cluster. The time series of standardized flows for each site in each cluster were averaged to produce a cluster average of standardized flows. Subsequently, the time series of standardized flows for each site then were correlated with each cluster average time series and each site was ultimately assigned to the cluster with which it had the highest correlation. We obtained a representative time series for each cluster by computing the mean of flows at gauges in each cluster for each year. This “cluster-mean flow” represents the dominant temporal patterns and cycles of peak and seasonal maximum flow variability within each region.

### Wavelet transform of cluster-mean flows

We used the R package *biwavelet*^41^ to compute the wavelet transform of the cluster-mean flows of annual peak flows and seasonal maximum flows, as well as the 3-year moving average of cluster-mean flows. Plots of the spectra of wavelet transforms for the annual peaks and maximum seasonal flows (OND, JFM, AMJ, and JAS) are shown in Supporting Figs 2–6, respectively. The same spectra of the 3-year moving averages for annual peaks and maximum seasonal flows (OND, JFM, AMJ, and JAS) are shown in Supporting Figs 7–11, respectively.

### Climate teleconnections

To evaluate regional patterns of peak and seasonal maximum flows and global-scale climatic drivers, we correlated the cluster-mean flows with each climate index to identify teleconnections related to SST using Pearson correlation. Significance was defined at the 95% confidence level (*p* < 0.05). We correlated the maximum seasonal streamflow with the concurrent seasonal mean of the climate indices. The standardized annual peaks were correlated with the mean of each climate index over the months OND, JFM, AMJ, and JAS of the same water year. The seasonal maximum flows were correlated with the mean of the time series for the concurrent months. To evaluate the strength of the correlations of the cluster-mean flow, we also correlated the individual time series of standardized annual peak and seasonal maximum streamflows at each gauge with the climate indices, and the cluster-mean flow with mean water-year SST.

### Evaluating the relations of clusters and climate indices

To assess the relations between floods and climate drivers, we correlated the cluster-mean flows with climate indices. The correlation patterns of the individual gauges (Fig. S1) with the climate indices matched the regions of the clusters for several, but not all teleconnections, and the percentage of the gauges with significant correlations in a cluster varies (Tables 1 and S1). For example, the correlation coefficient between the JFM cluster 2 mean and MEI is 0.50 (*p* = 2E-4, Table S1) and 76% of the gauges have significant correlation, whereas the correlation between cluster 3 for annual peaks mean and JFM MEI is 0.61 (*p* = 3E-6, Table 2) and fewer gauges (36%) in the cluster have significant correlations. Thus, the question remains about how well the clusters represent the patterns of teleconnections.

To evaluate the clusters in terms of how well they represent a climatic influence over a region, we used field significance^64^ to assess the collective significance of the spatial pattern of correlations at gauges within each cluster. That is, we assessed whether the number of significant correlations within a cluster is significant, which provides some measure of whether the correlation pattern as a cohesive group is significant and not substantially affected by serial correlation. To estimate the field significance of the relations between each cluster and climate index, we calculated a large set of correlations between the streamflow time series, reordered through time using permutation, with the climate indices (not reordered). Then we calculated the percentage of gauges with significant correlation (*p* < 0.05). We reordered the time series 10,000 times to obtain a null distribution of the percentage of gauges that passed the significance test. Field significance for each cluster and climate relation was satisfied if the actual percentage of significant gauges in the data was matched in less than 5% of the randomized tests. The clusters that passed the field significance test are indicated in Tables 1 and S1 by the percentage of significant gauges in bold text. Field significance was generally satisfied for cases of significant correlation between the cluster means and climate indices, meaning the clusters are robust for representing the patterns of teleconnections. In cases where the correlation of the cluster-mean time series and time series was significant, but field significance was not achieved (for example, peaks cluster 2 and AO), the cluster-mean time series was likely dominated by a few gauges of higher correlation with the climate index. The opposite case of field significance but no significant correlation by the cluster mean suggests that the cluster is robust in that it represents a region with a weak climate teleconnection.

## Supplementary information

Supplementary Info

## Data Availability

Peak flow data for the gauges in this study are available for download from the U.S. Geological Survey National Water Information System (NWIS) at waterdata.usgs.gov and in U.S. Geological Survey data releases^65,66^.
