# Supplementary material for: Seasonality of climatic drivers of flood variability in the conterminous United States
Source: Sci Rep. 2019 Oct 25;9:15321. doi: 10.1038/s41598-019-51722-8 (PMC6814712; doi:10.1038/s41598-019-51722-8)
Supplement: Supplementary file 1 — Supplementary Info [file 41598_2019_51722_MOESM1_ESM.docx]

Seasonality of climatic drivers of flood variability in the conterminous United States

Jesse E. Dickinson*^1^, Tessa Harden^2^, Gregory J. McCabe^3^

^1^ U.S. Geological Survey, Tucson, Arizona, USA, jdickins@usgs.gov

^2^ U.S. Geological Survey, Portland, Oregon, USA, tharden@usgs.gov

^3^ U.S. Geological Survey, Denver, Colorado, USA, gmccabe@usgs.gov

# Supplementary information

*Table S1:* Correlations between the mean time series for the peaks and seasonal-maximum flow clusters and climate indices, and the percentage of gages in each cluster with significant correlation with the indices (bold if passed the field significance test). Correlations of the mean time series that are significant at the 95% confidence level are in bold and outlined cells. Correlations that are significant at the 99% confidence level are shown as larger text in bold in outlined cells. The ‘--‘ indicates no correlation test.

|  |  | cluster | | | | | | | | |
| --- | --- | --- | --- | --- | --- | --- | --- | --- | --- | --- |
|  | climate index | 1 | 2 | 3 | 4 | 5 | 6 | 7 | 8 | 9 |
| OND | MEI | -0.20 8% | -0.13 5% | 0.19 4% | 0.14 9% | 0.22 11% | -0.05 4% | -0.23 8% | -- | -- |
|  | PDO | **-0.36 50%** | 0.02 2% | 0.13 4% | -0.06 11% | 0.17 1% | -0.01 1% | -0.02 0% | -- | -- |
|  | PNA | -0.10 0% | -0.09 2% | -0.23 0% | -0.13 3% | -0.15 10% | **-0.39 29%** | -0.04 0% | -- | -- |
|  | AMO | **0.28 28%** | 0.15 0% | -0.23 15% | 0.12 11% | **-0.33 22%** | -0.16 7% | 0.15 11% | -- | -- |
|  | NAO | -0.02 0% | **-0.34 48%** | 0.22 **29%** | 0.22 11% | 0.14 9% | 0.01 1% | -0.26 19% | -- | -- |
|  | AO | 0.17 6% | **-0.29 43%** | 0.07 12% | 0.26 20% | 0.14 7% | 0.09 1% | -0.14 6% | -- | -- |
| JFM | MEI | -0.13 6% | **0.50 76%** | 0.01 0% | 0.10 4% | **0.44 58%** | -0.06 13% | **0.48 42%** | 0.27 **42%** | **0.28 35%** |
|  | PDO | -0.11 8% | 0.22 2% | 0.12 7% | 0.15 0% | 0.25 15% | -0.11 3% | 0.03 2% | 0.16 6% | 0.20 15% |
|  | PNA | -0.28 **26%** | 0.20 5% | 0.07 2% | 0.01 11% | **0.30** 12% | -0.20 18% | 0.01 13% | 0.05 4% | **0.32 35%** |
|  | AMO | -0.03 0% | 0.09 2% | 0.10 7% | -0.02 4% | 0.09 4% | 0.05 3% | -0.13 2% | -0.03 1% | -0.07 3% |
|  | NAO | 0.21 3% | -0.13 2% | **0.34 35%** | -0.08 0% | -0.08 0% | -0.12 3% | -0.06 2% | -0.16 6% | -0.09 8% |
|  | AO | 0.15 0% | -0.19 5% | 0.14 16% | -0.12 0% | -0.05 0% | -0.02 0% | -0.07 0% | -0.13 6% | -0.09 10% |
| AMJ | MEI | **-0.34 31%** | -0.04 6% | **0.40 34%** | 0.24 17% | 0.18 4% | -- | -- | -- | -- |
|  | PDO | **-0.32 24%** | 0.03 1% | **0.28 19%** | 0.10 1% | 0.11 4% | -- | -- | -- | -- |
|  | PNA | -0.25 7% | 0.12 6% | **0.50 44%** | -0.12 8% | 0.12 2% | -- | -- | -- | -- |
|  | AMO | -0.25 17% | 0.11 8% | -0.15 10% | -0.24 15% | -0.05 4% | -- | -- | -- | -- |
|  | NAO | 0.01 3% | **-0.31** 11% | -0.09 0% | 0.10 8% | 0.11 4% | -- | -- | -- | -- |
|  | AO | -0.05 3% | **-0.31 26%** | **-0.34 30%** | 0.05 3% | 0.07 4% | -- | -- | -- | -- |
| JAS | MEI | -0.08 4% | -0.14 1% | -0.00 1% | -0.09 4% | -0.14 2% | -- | -- | -- | -- |
|  | PDO | -0.04 1% | -0.09 7% | 0.25 9% | -0.05 5% | **-0.34** 13% | -- | -- | -- | -- |
|  | PNA | -0.05 0% | -0.03 1% | 0.22 9% | -0.14 6% | 0.08 0% | -- | -- | -- | -- |
|  | AMO | -0.26 10% | -0.20 5% | -0.15 8% | 0.03 2% | 0.27 16% | -- | -- | -- | -- |
|  | NAO | 0.25 9% | 0.19 9% | -0.08 4% | **0.42 34%** | 0.01 9% | -- | -- | -- | -- |
|  | AO | -0.08 6% | 0.07 2% | **-0.39 26%** | **0.28 15%** | 0.03 11% | -- | -- | -- | -- |


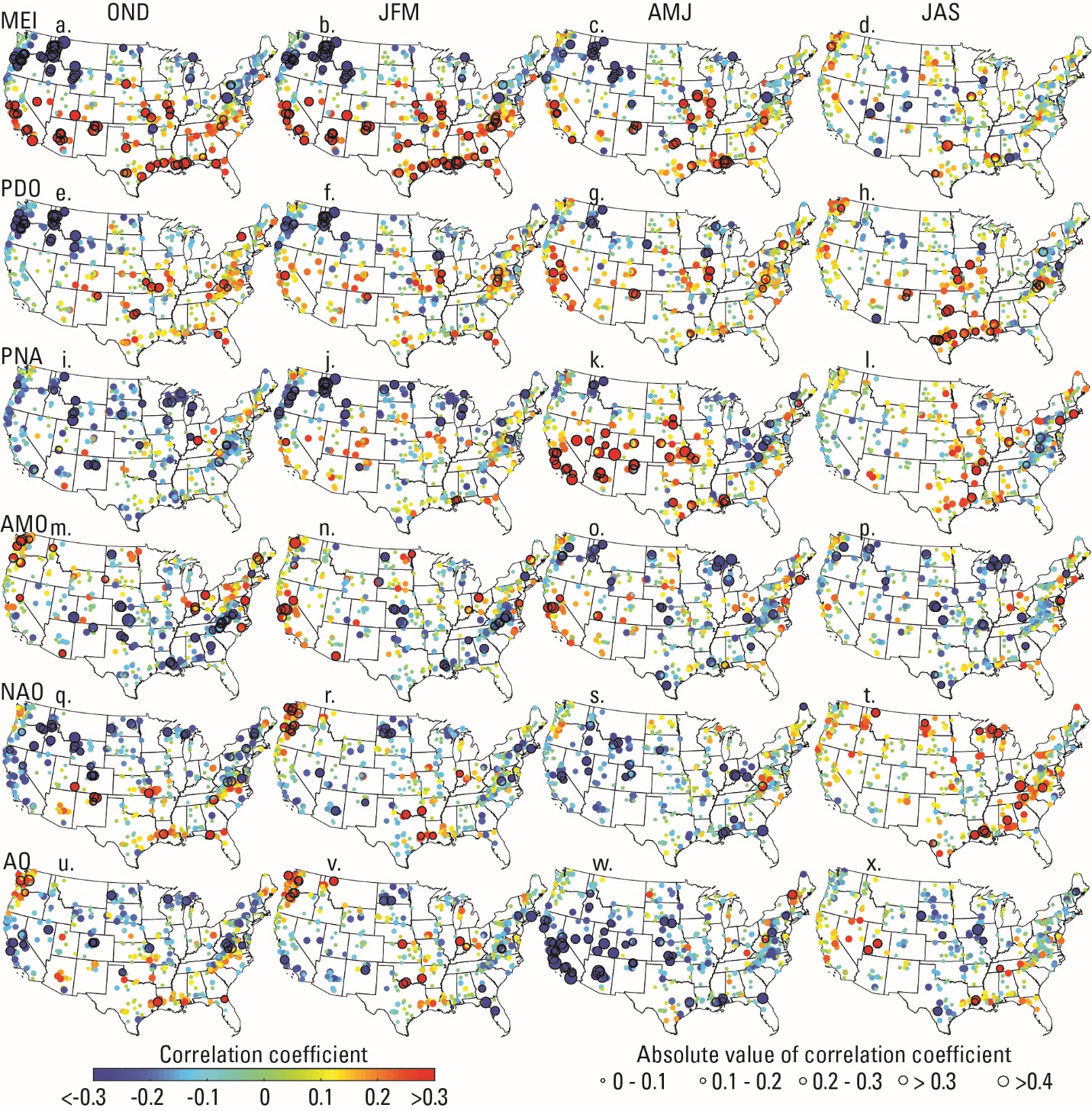


Figure S1: Correlation of seasonal maximum streamflow, arranged by the season in the columns, with climate indices arranged in rows in order from top to bottom of MEI (a through d), PDO (e through h), PNA (i through l), AMO (m through p), NAO (q through t), and AO (u through x). The color and size of the marker indicates the value of the correlation coefficient. Markers with dark outlines indicate significance at *p* < 0.05.


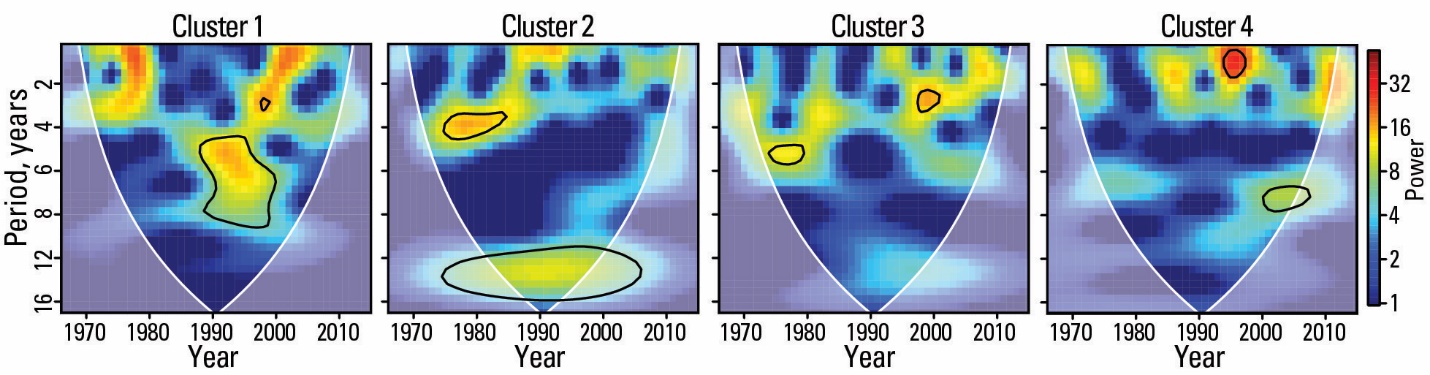


Figure S2: Wavelet analysis of the peaks cluster-mean flows. Areas in red indicate larger power over the band of periods through time. Areas that are circled indicate significance at *p* < 0.05.


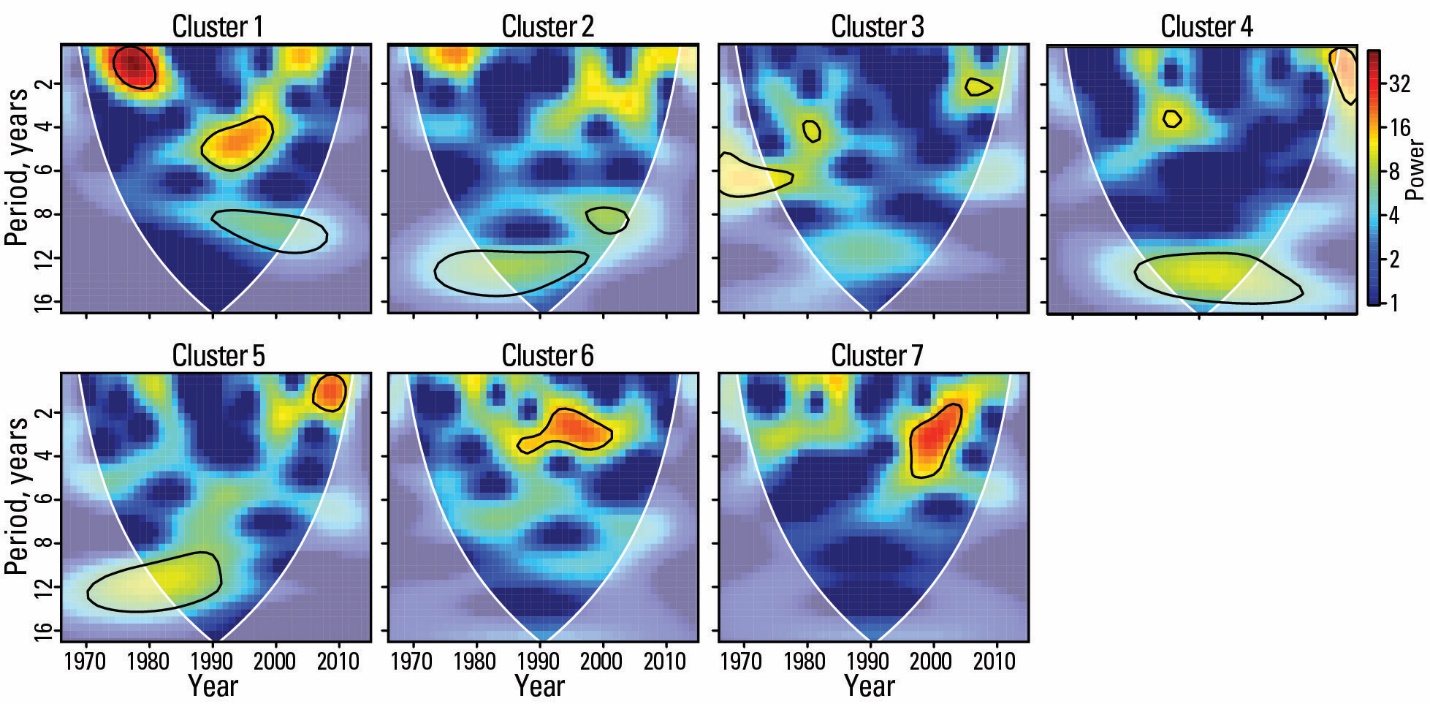


Figure S3: Wavelet analysis of the OND cluster-mean flows. Areas in red indicate larger power over the band of periods through time. Areas that are circled indicate significance at *p* < 0.05.


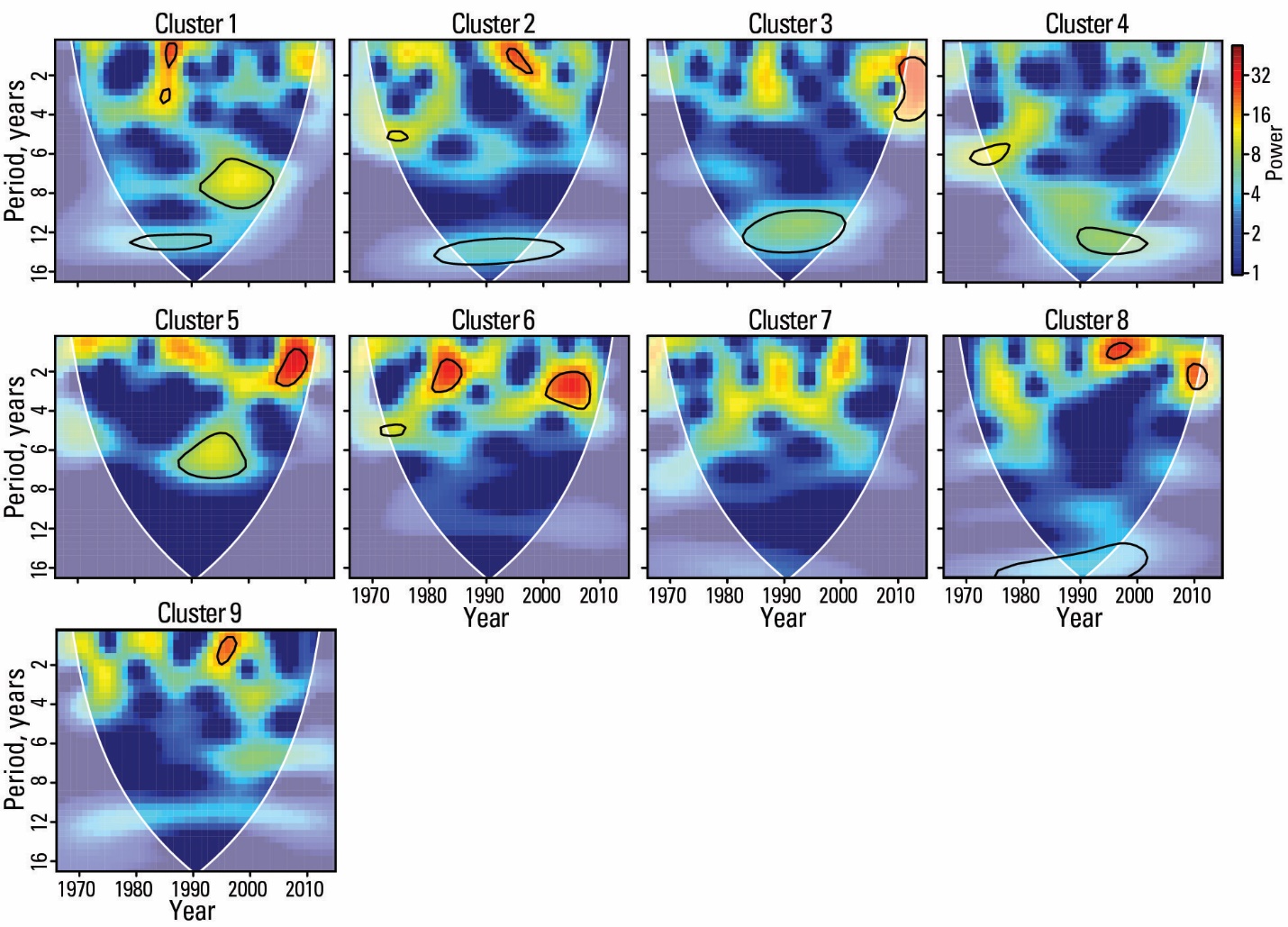


Figure S4: Wavelet analysis of the JFM cluster-mean flows. Areas in red indicate larger power over the band of periods through time. Areas that are circled indicate significance at *p* < 0.05.


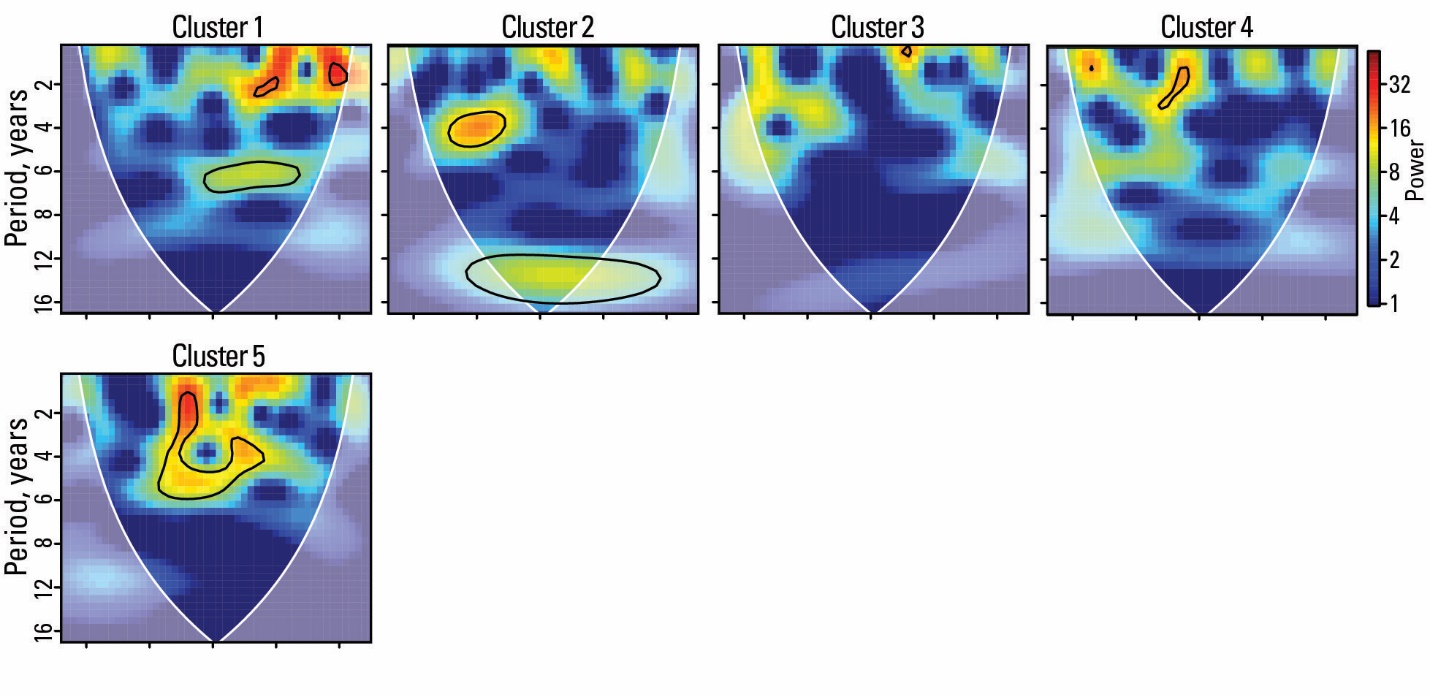


Figure S5: Wavelet analysis of the AMJ cluster-mean flows. Areas in red indicate larger power over the band of periods through time. Areas that are circled indicate significance at *p* < 0.05.


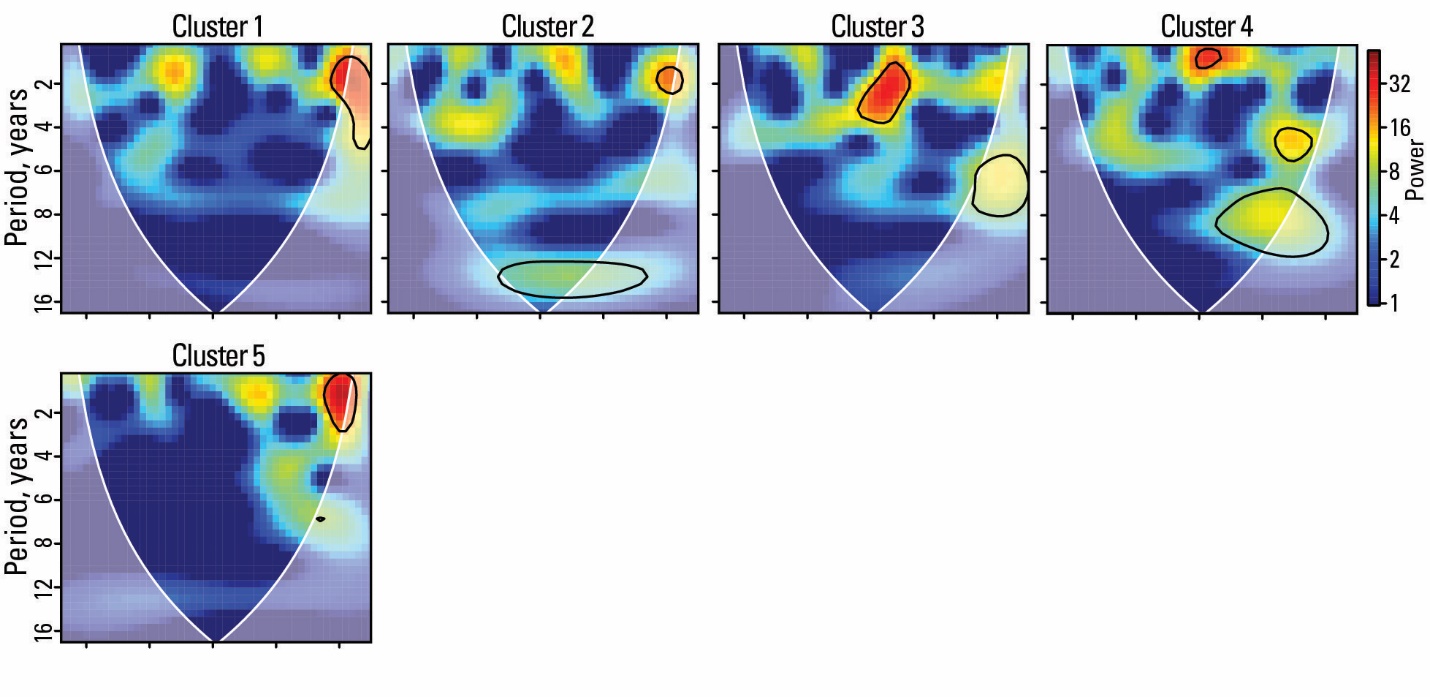


Figure S6: Wavelet analysis of the JAS cluster-mean flows. Areas in red indicate larger power over the band of periods through time. Areas that are circled indicate significance at *p* < 0.05.


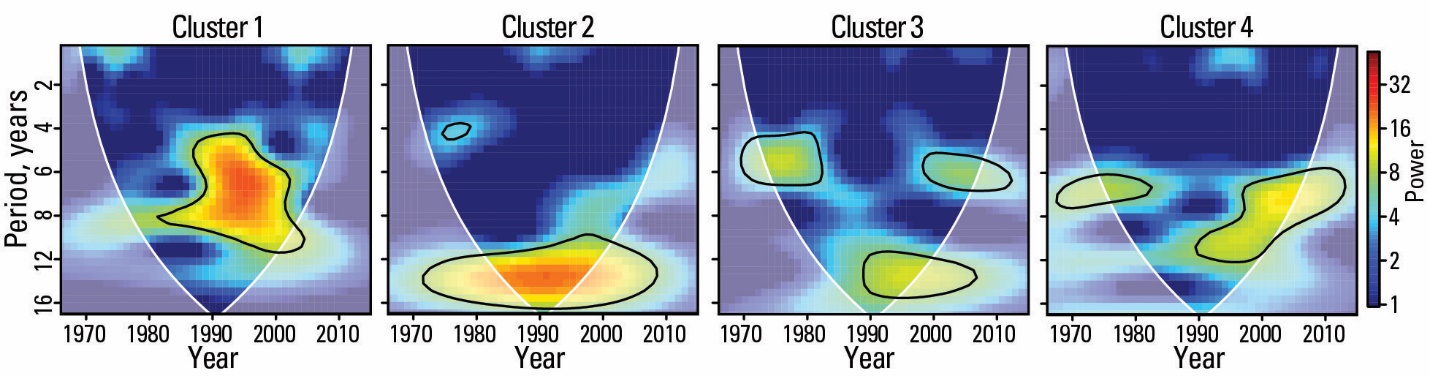


Figure S7: Wavelet analysis of the 3-year moving average of the peaks cluster-mean flows. Areas in red indicate larger power over the band of periods through time. Areas that are circled indicate significance at *p* < 0.05.


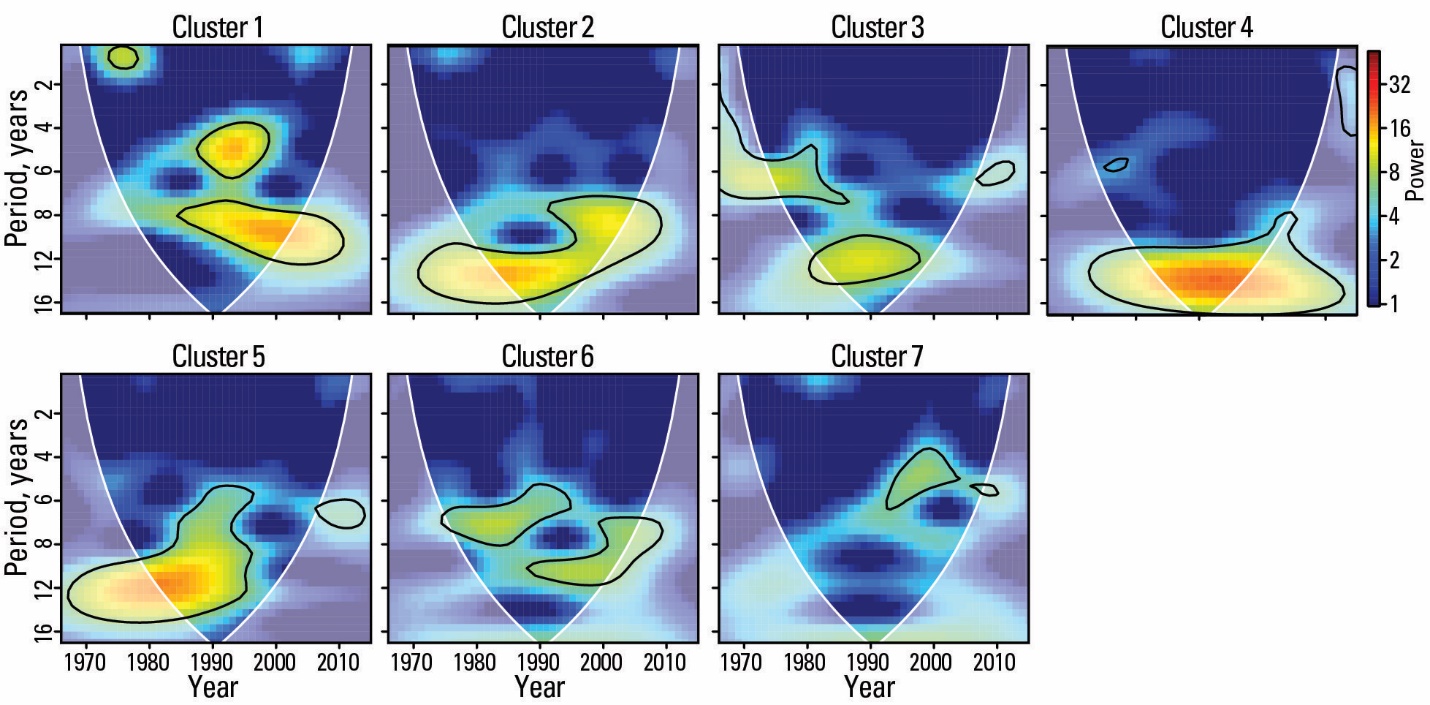


Figure S8: Wavelet analysis of the 3-year moving average of the OND cluster-mean flows. Areas in red indicate larger power over the band of periods through time. Areas that are circled indicate significance at *p* < 0.05.


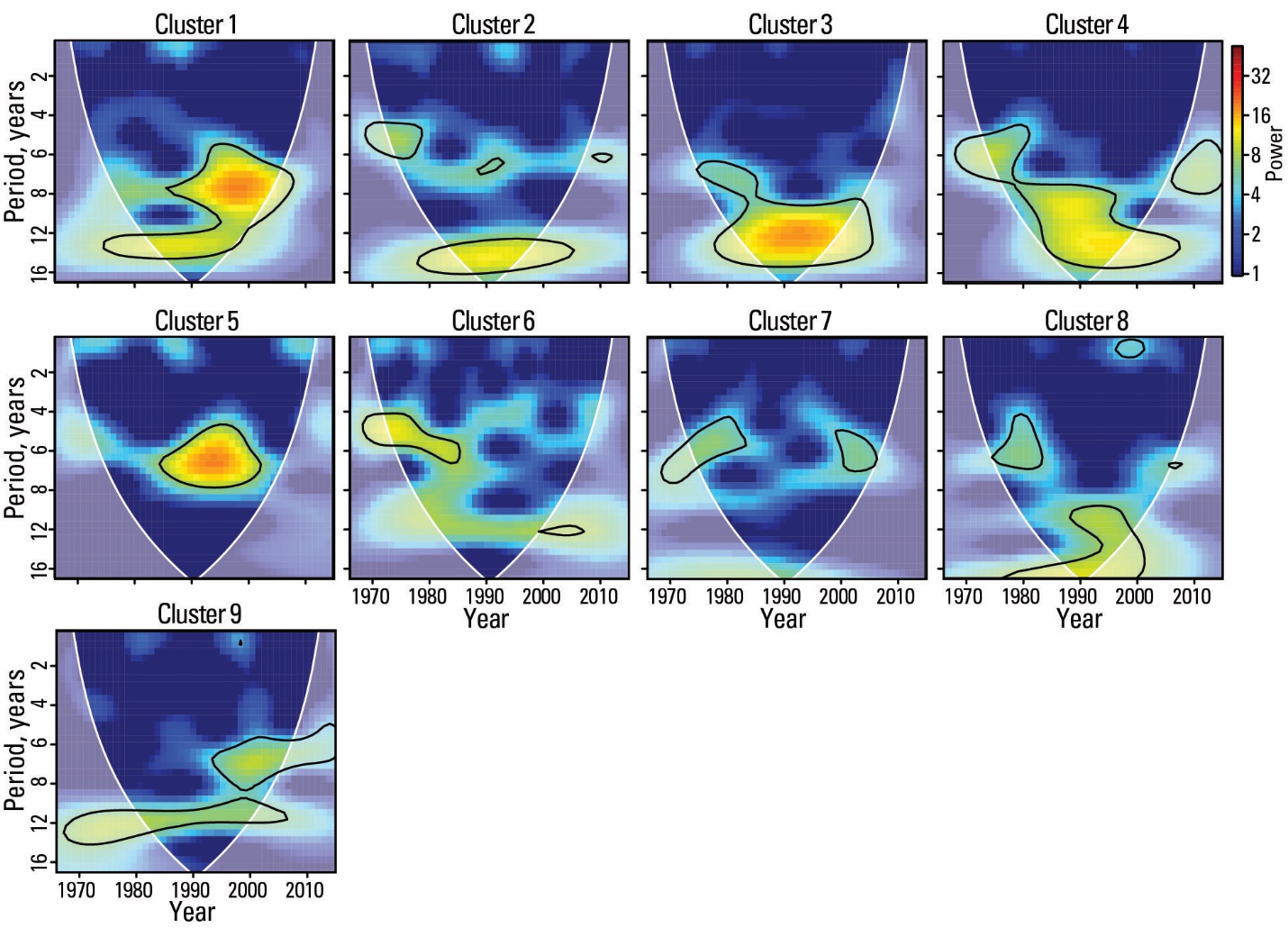


Figure S9: Wavelet analysis of the 3-year moving average of the JFM cluster-mean flows. Areas in red indicate larger power over the band of periods through time. Areas that are circled indicate significance at *p* < 0.05.


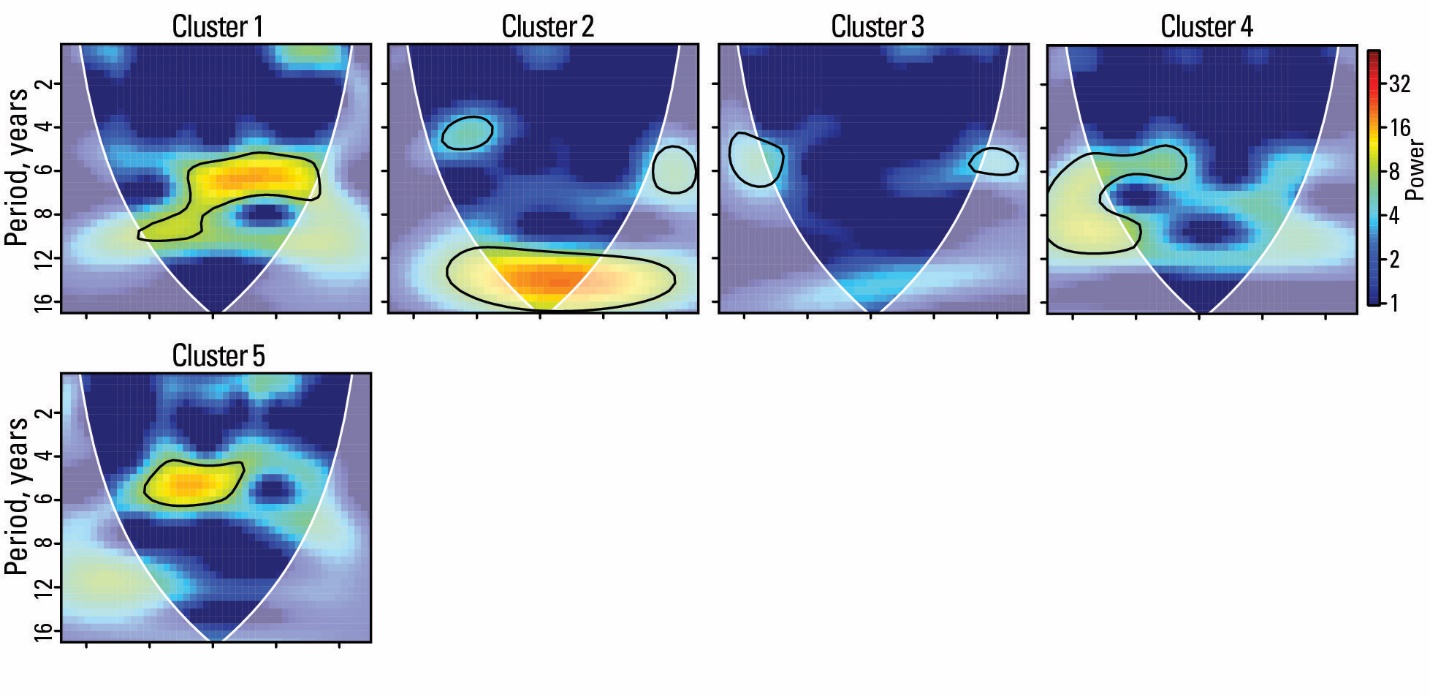


Figure S10: Wavelet analysis of the 3-year moving average of the AMJ cluster-mean flows. Areas in red indicate larger power over the band of periods through time. Areas that are circled indicate significance at *p* < 0.05.


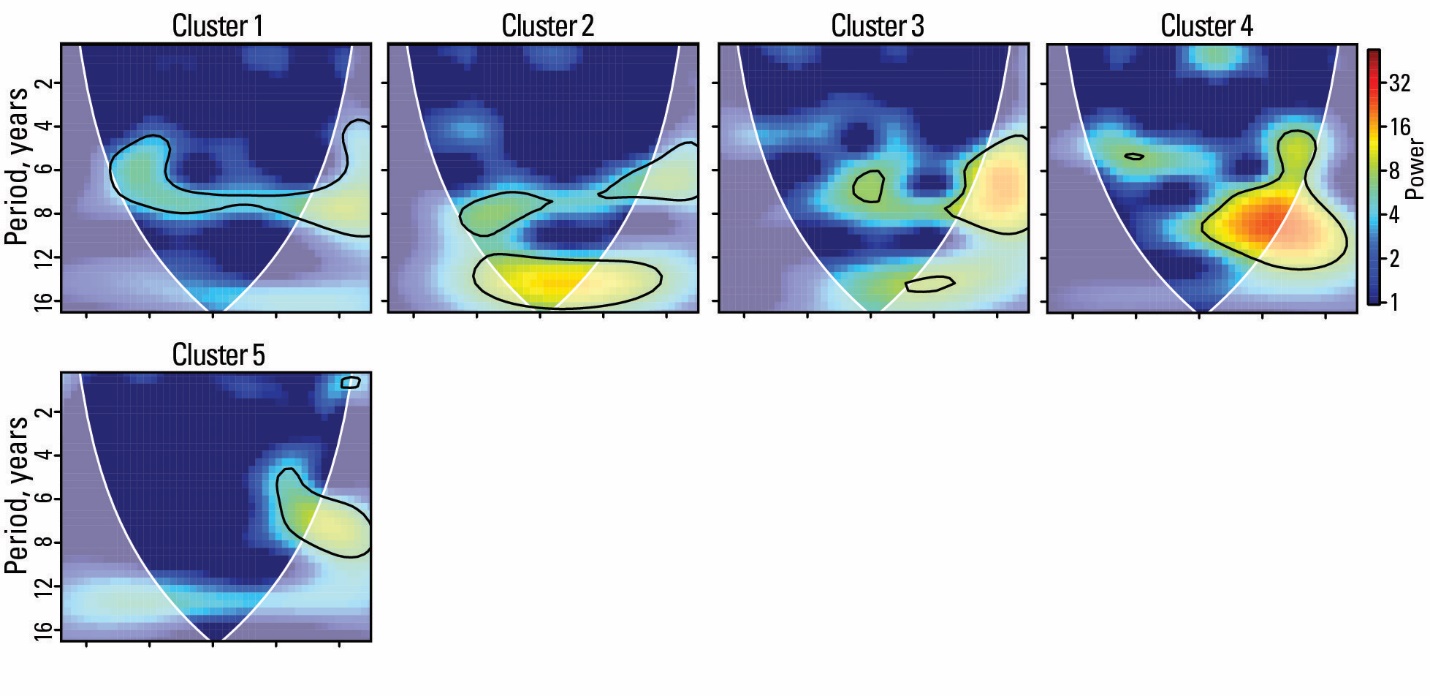


Figure S11: Wavelet analysis of the 3-year moving average of the JAS cluster-mean flows. Areas in red indicate larger power over the band of periods through time. Areas that are circled indicate significance at *p* < 0.05.


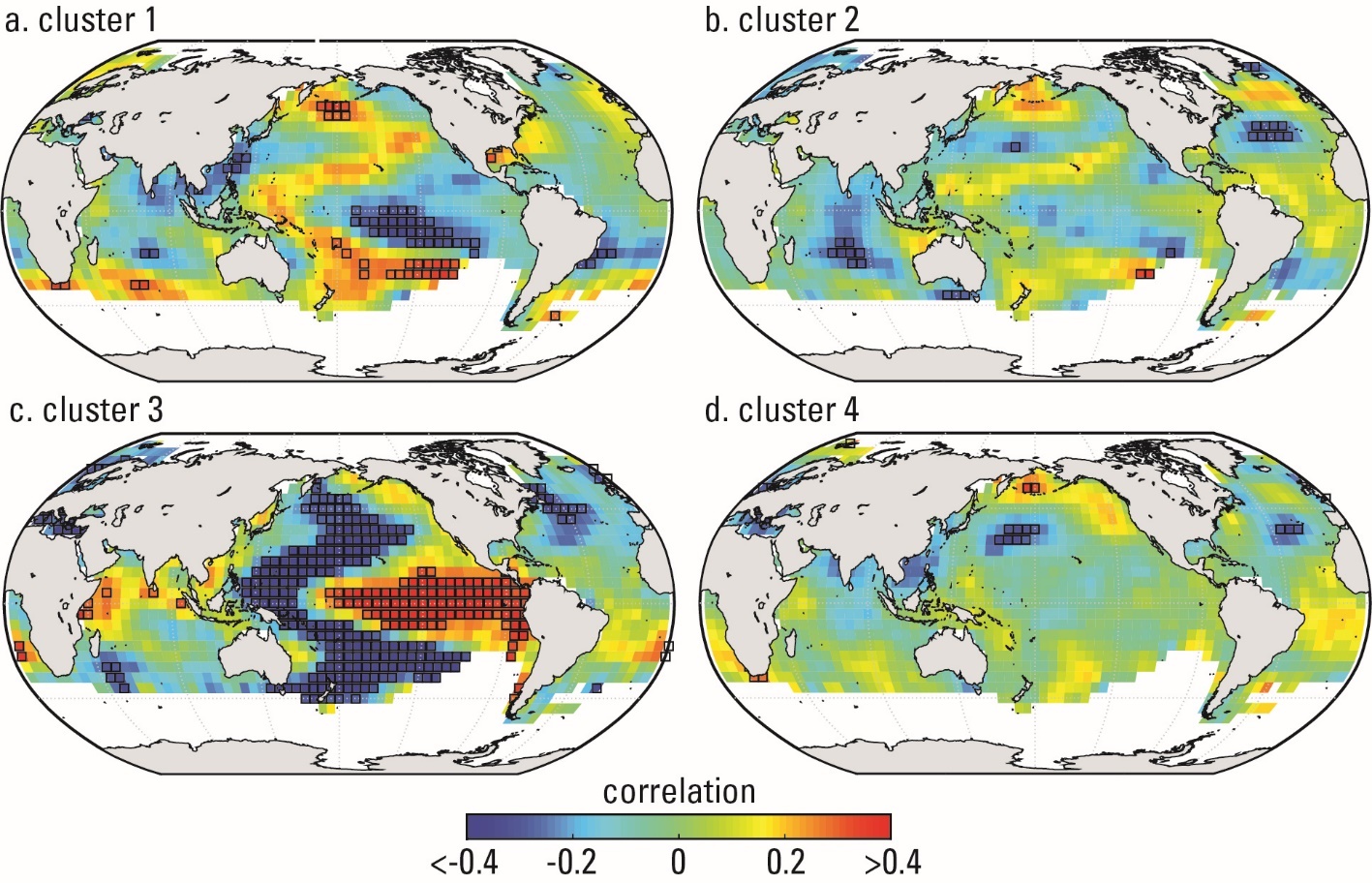


Figure S12: Correlation between the mean time series for the peaks clusters and mean annual (water year of Oct-Sep) sea surface temperature from 1966 to 2015. A square outline indicates significance at *p* < 0.05.


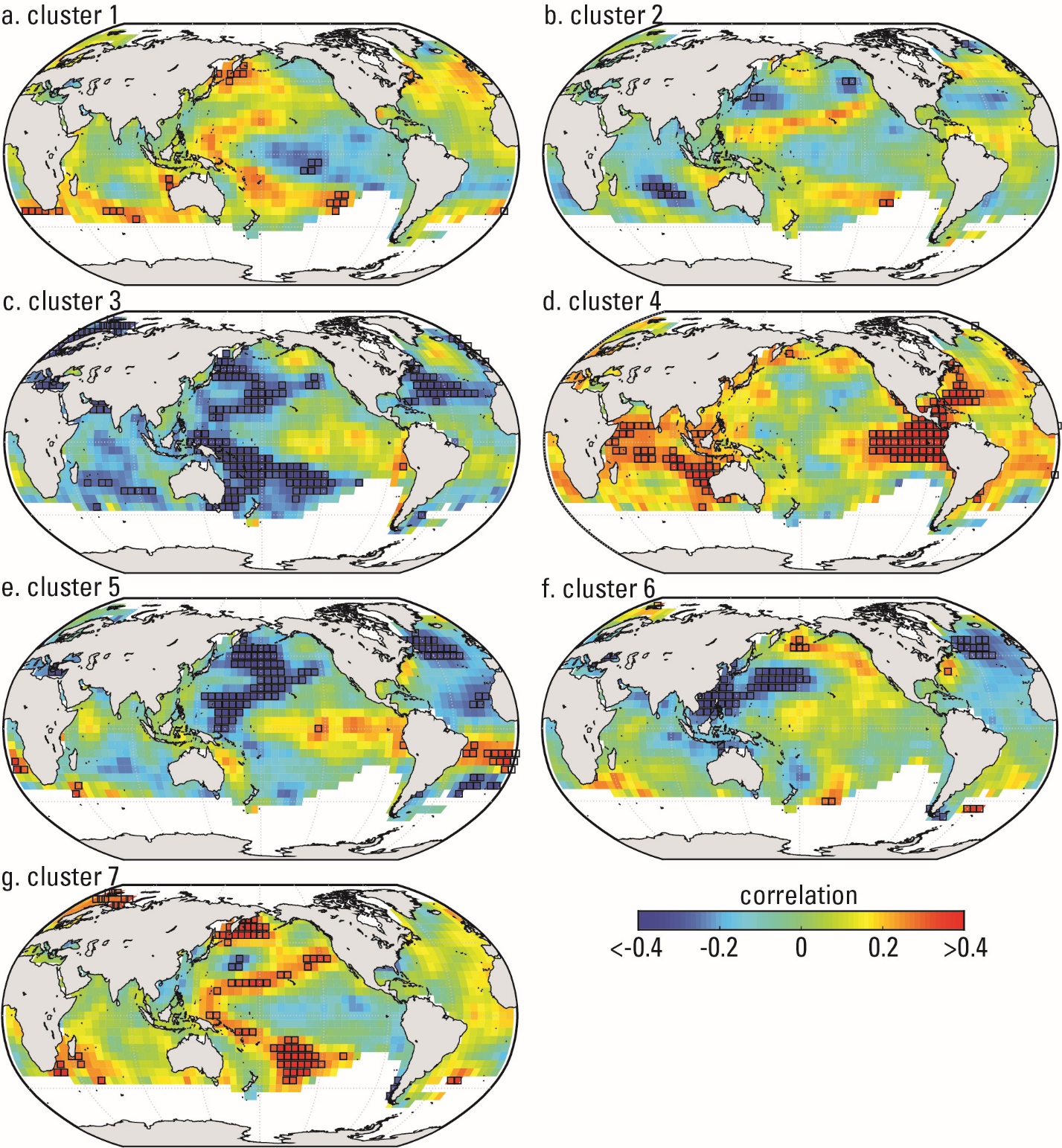


Figure S13: Correlation between the mean time series for the OND clusters and mean annual (water year of Oct-Sep) sea surface temperature from 1966 to 2015. A square outline indicates significance at *p* < 0.05.


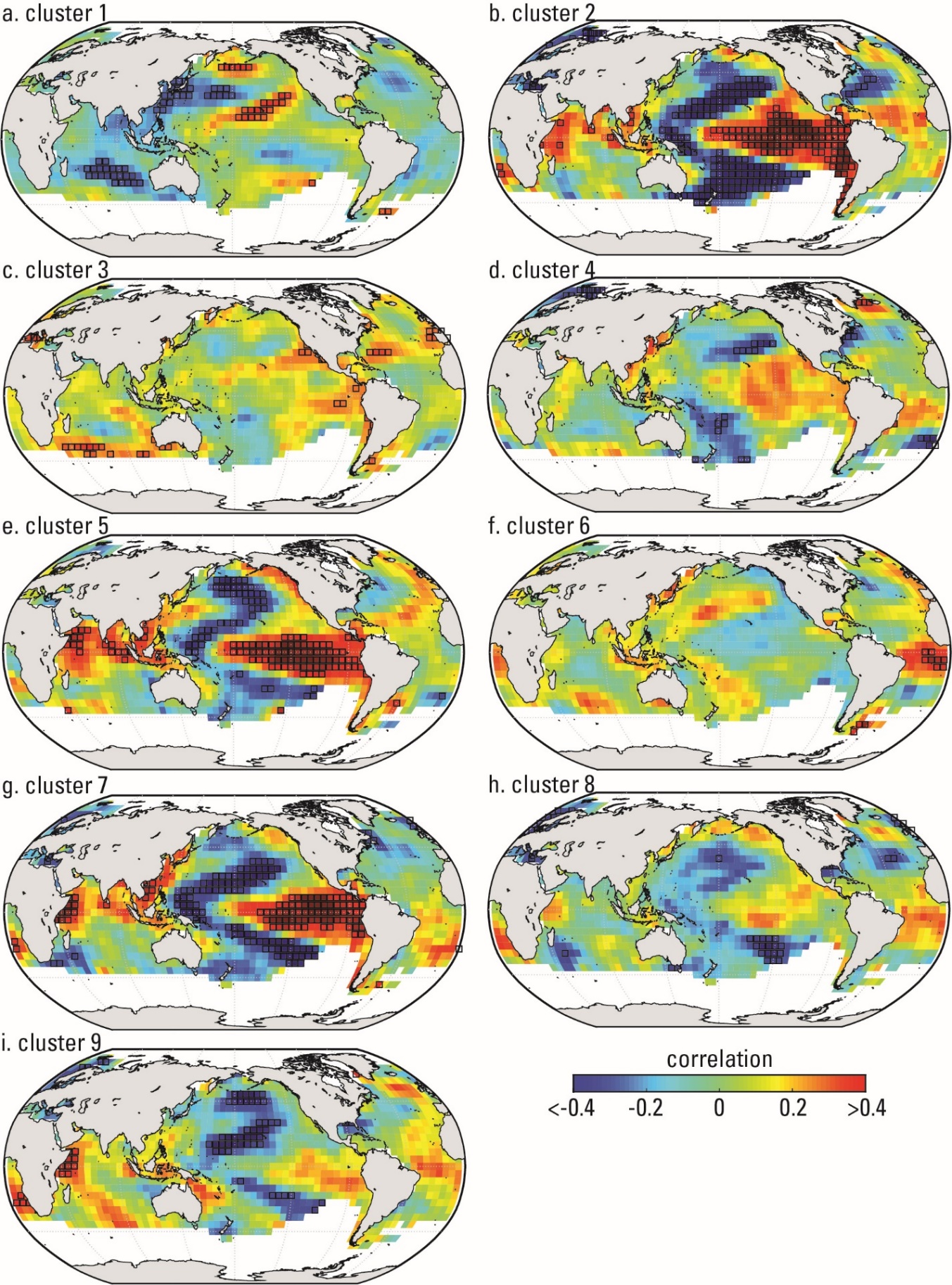


Figure S14: Correlation between the mean time series for the JFM clusters and mean annual (water year of Oct-Sep) sea surface temperature from 1966 to 2015. A square outline indicates significance at *p* < 0.05.


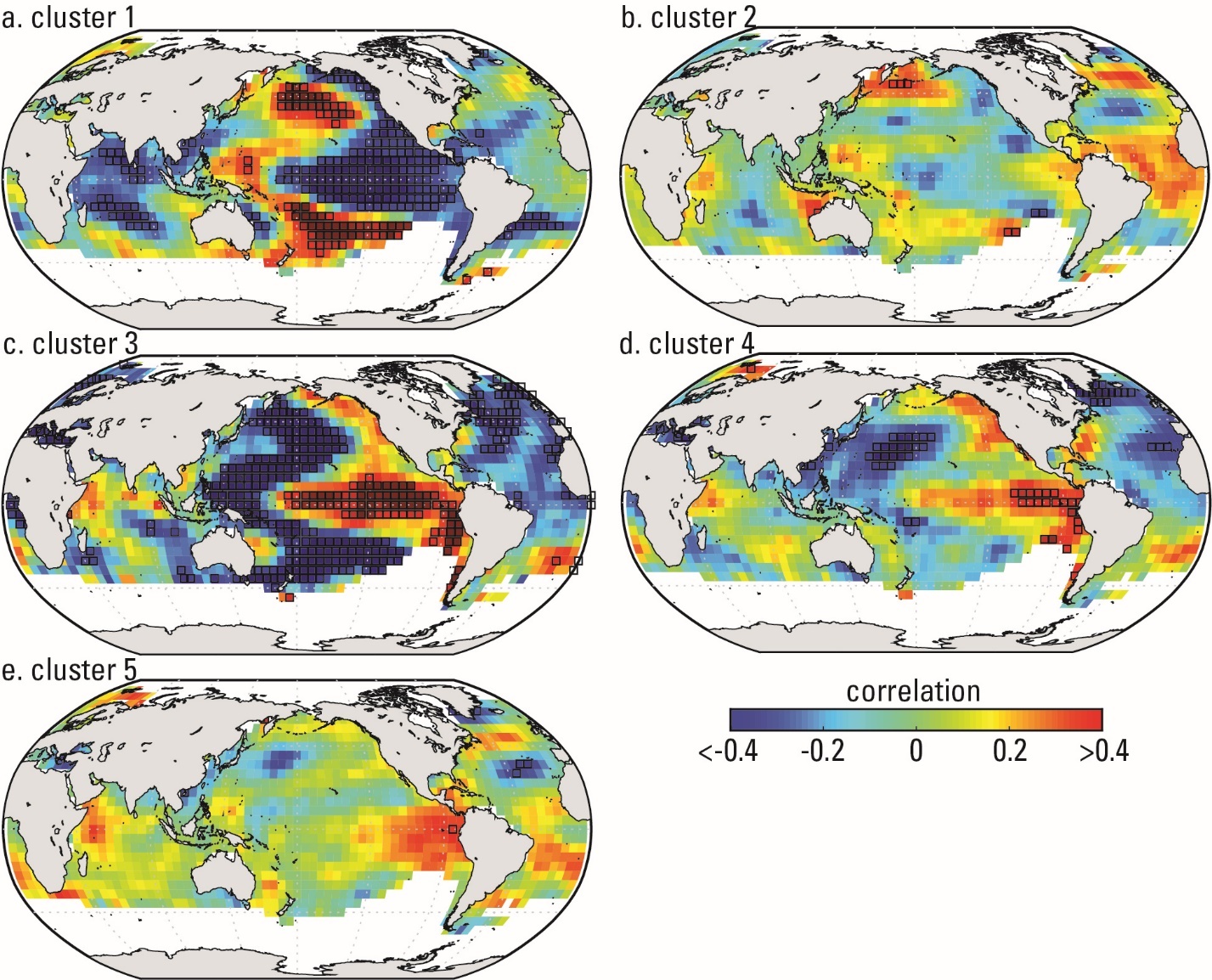


Figure S15: Correlation between the mean time series for the AMJ clusters and mean annual (water year of Oct-Sep) sea surface temperature from 1966 to 2015. A square outline indicates significance at *p* < 0.05.


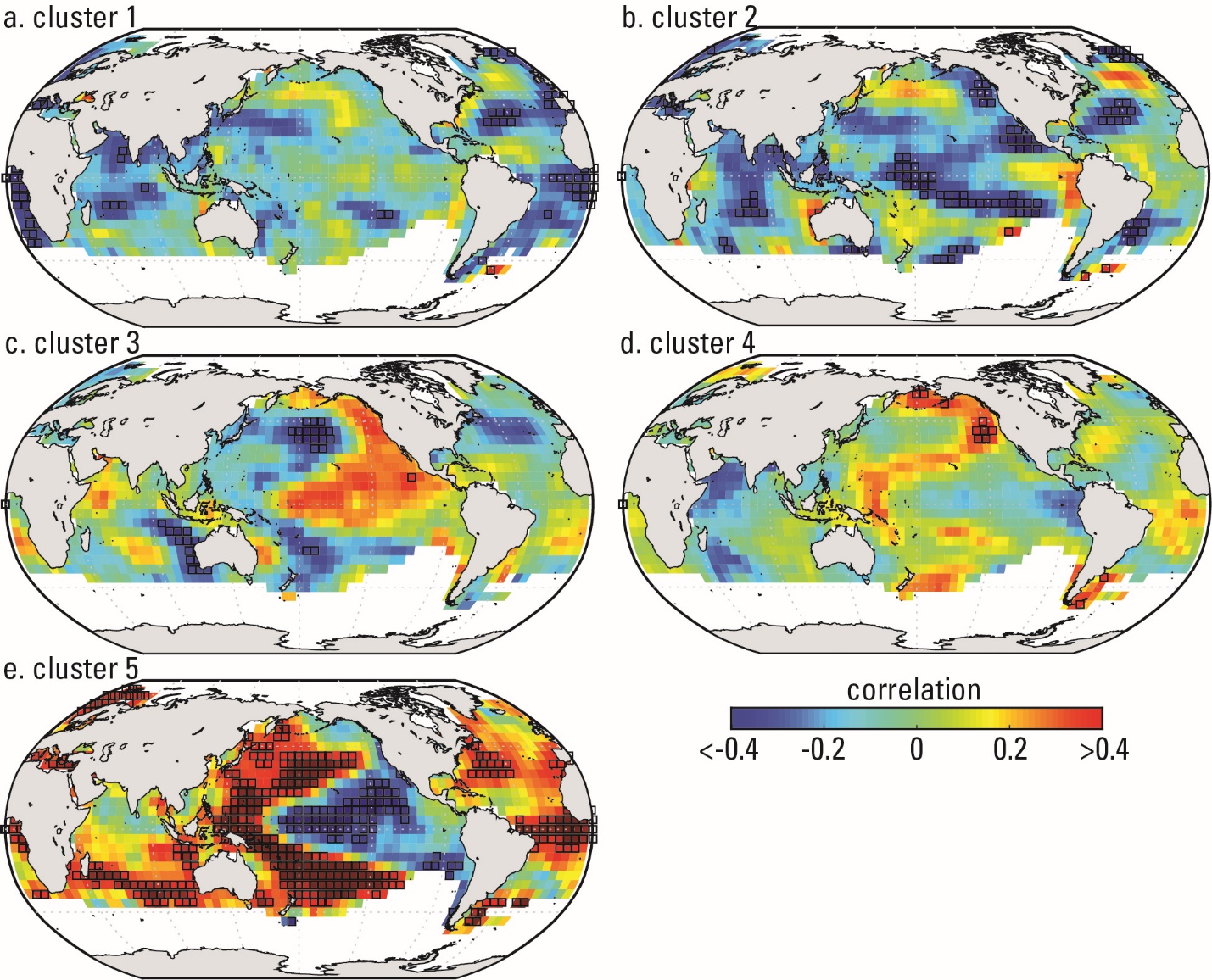


Figure S16: Correlation between the mean time series for the JAS clusters and mean annual (water year of Oct-Sep) sea surface temperature from 1966 to 2015. A square outline indicates significance at *p* < 0.05.
